# Understanding the root of the problem for tackling pea root rot disease

**DOI:** 10.3389/fmicb.2024.1441814

**Published:** 2024-10-24

**Authors:** Nicolas Karl Trenk, Alba Pacheco-Moreno, Sanu Arora

**Affiliations:** Department of Biochemistry and Metabolism, John Innes Centre, Norwich Research Park, Norwich, United Kingdom

**Keywords:** pea cultivation, sustainable agriculture, root rot, soil microbiome, genetic resistance, diagnostic tools

## Abstract

Pea (*Pisum sativum*), a crop historically significant in the field of genetics, is regaining momentum in sustainable agriculture due to its high protein content and environmental benefits. However, its cultivation faces significant challenges from root rot, a complex disease caused by multiple soil-borne pathogens prevalent across most pea growing regions. This disease leads to substantial yield losses, further complicated by the dynamic interactions among pathogens, soil conditions, weather, and agricultural practices. Recent advancements in molecular diagnostics provide promising tools for the early and precise detection of these pathogens, which is critical for implementing effective disease management strategies. In this review, we explore how the availability of latest pea genomic resources and emerging technologies, such as CRISPR and cell-specific transcriptomics, will enable a deeper understanding of the molecular basis underlying host-pathogen interactions. We emphasize the need for a comprehensive approach that integrates genetic resistance, advanced diagnostics, cultural practices and the role of the soil microbiome in root rot. By leveraging these strategies, it is possible to develop pea varieties that can withstand root rot, ensuring the crop's resilience and its continued importance in global agriculture.

## 1 Introduction

Pea (*Pisum sativum*) is an annual cool-season crop constituting the second most important grain legume worldwide (Sari et al., 2021). From being used by Mendel to lay the groundwork for modern genetics, this crop is once again gaining popularity. With the increasing interest in healthy diets and awareness about the negative environmental impact and ethical implications of animal-sourced protein, pea serves as a high-quality alternative due to its richness in protein, healthy starch, and fiber, complemented by a favorable amino acid profile and low allergenicity (Anishkumar et al., 2022; Kouris-Blazos and Belski, 2016). Peas provide environmental benefits through reduced eutrophication, ranking among the lowest in crop land usages per 100 g protein produced. They also provide carbon sequestration, regenerate nutrient-deficient soils, and increase the nitrogen-use efficiency of other crops in rotation (Anishkumar et al., 2022; Bedoussac et al., 2015; Madsen et al., 2022). With rising energy prices and shortages strongly influencing fertilizer availability, low-input crops have become particularly desirable for farmers. This is further reinforced by the rising demand for plant protein. In 2020, the global plant-based protein market amounted to USD 10.3 billion, with a projected growth to USD 85 billion by 2030 (Sha and Xiong, 2020). Taken together, these factors make pea an indispensable choice for sustainable agriculture and the food industry.

**Figure 1:**
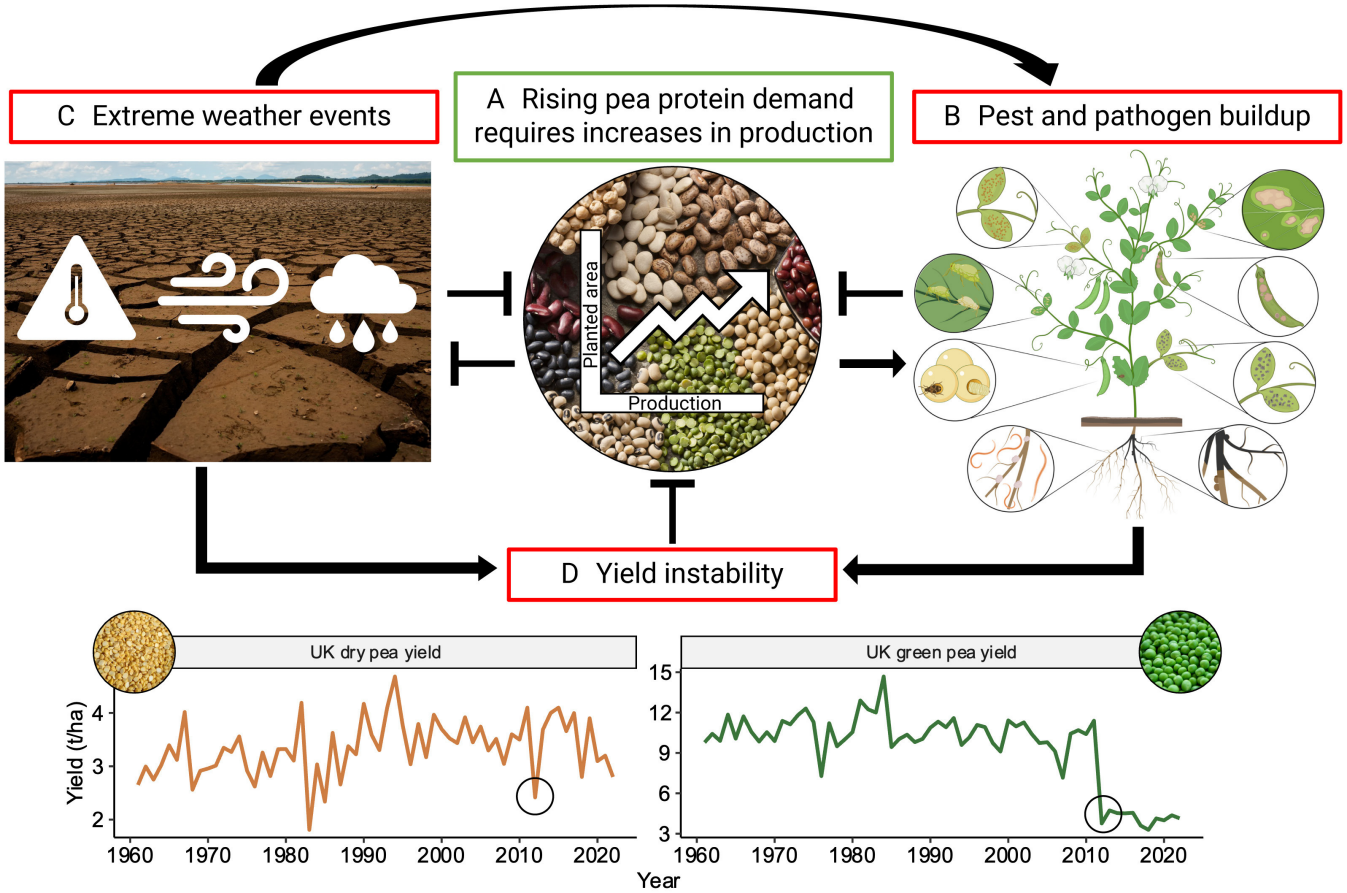
Factors influencing pea production and their interactions. This figure illustrates the interconnected factors impacting pea production, where sharp arrows (→) denote reinforcement and blunt arrows (⊥) indicate inhibition. **(A)** Legumes such as pea are an indispensable part of sustainable agriculture which aims to mitigate the negative effects of climate change. The increasing demand for plant-based protein necessitates a rise in pea production, currently achieved mainly through expanding growing area and employing narrow crop rotations. **(B)** However, their intensive cultivation leads to the build-up of pests and pathogens, which include root rot, root nematodes, bruchids, aphids and related viral infections, rust, bacterial blight, Ascochyta blight and downy mildew (illustrated clockwise from the bottom right). Disease figure created in Biorender. **(C)** Furthermore, climate change exacerbates biotic stresses triggered through increased extreme weather events. In addition, the pea crop is significantly impacted by heat stress, droughts, lodging and waterlogging challenges. **(D)** All these factors contribute to yield instability, for instance in the year 2012 (as indicated by black circles), particularly wet conditions in the UK exacerbated biotic yield losses [data from FAOSTAT (2024)]. This instability presents an economic risk to pea growers, impeding further increases in pea production.

Despite this substantial market growth potential, the increased production of peas worldwide is not generally associated with yield enhancements or improved cultivars, but rather with an increase in harvested area, suggesting an underutilization of their full genetic potential (Figure 1A) (Foyer et al., 2016). This limitation is further exacerbated by the erratic pattern of climate change-triggered extreme weather events. Peas are particularly vulnerable to heat and drought stress, which negatively impact their yield stability (Figures 1C, D) (Bueckert et al., 2015). Consequently, pea growers face uncertainty in making critical decisions such as sowing date, which can result in delayed flowering and an increased risk of pathogen infections caused by high temperatures (Foyer et al., 2016). Among these pathogens, soil-borne diseases present an especially formidable challenge for pea growers, as the severity of infections increases sharply with delays in flowering (Kalil and Wunsch, 2024). Furthermore, climate change exacerbates the severity of root rot as warmer temperatures and increased periods of drought followed by intense rainfall create ideal conditions for the proliferation and spread of these pathogens (Sharma et al., 2022).

Root rot is the most damaging soil disease affecting pea production globally, particularly in fields across North America, Northern Europe, England, Australia and New Zealand (Bodah, 2017). It is caused by a complex of pathogens which colonizes the plant's root system. These pathogens impair the plant's overall growth by disrupting nutrient and water uptake, leading to symptoms such as wilting, yellowing of leaves and stunted growth. In severe cases, it can lead to premature death of plants, resulting in substantial reductions in both yield and quality, and causing significant economic losses for growers (Figures 1B, D) (Kraft and Pfleger, 2001; Wu L. F. et al., 2022). The persistence of root rot pathogens in the soil organic matter or plant debris, often for several years, makes it difficult to manage and control them without any effective genetic and chemical treatments (Wille et al., 2019).

Currently, growers are relying on preventative measures, such as testing field soils for pathogens and avoiding contaminated areas (Gossen et al., 2016; Williamson-Benavides and Dhingra, 2021; Hossain et al., 2012), although these measures are not always sufficient. At the same time, disease pressure can be greatly affected by weather conditions, such as waterlogging, as different studies have shown that wet and compact soils are more conducive for root rot establishment (Scott, 1987; Allmaras et al., 2003). Given the current climate predictions, it is plausible to think that root rot will only become more prevalent, underscoring the need to address this issue from different perspectives. This review discusses the current knowledge and gaps in our understanding of pea root rot pathogens and highlights how rapidly expanding genomic resources and gene editing tools offer unique opportunities to address and mitigate this major constraint on pea cultivation. We also discuss advances in pathogen diagnostic tools based on molecular markers which are critical in early detection and management of root rot. The role of the soil beneficial microbiome, which antagonizes root rot pathogens or enhances plant resistance, could be leveraged to develop biocontrol strategies. By integrating these agronomic, genetic and molecular approaches, it is possible to develop management strategies to mitigate the impact of root rot in the face of a changing climate.

## 2 Root rot—A fight on multiple fronts with the soilborne adversary and its complications

**Figure 2:**
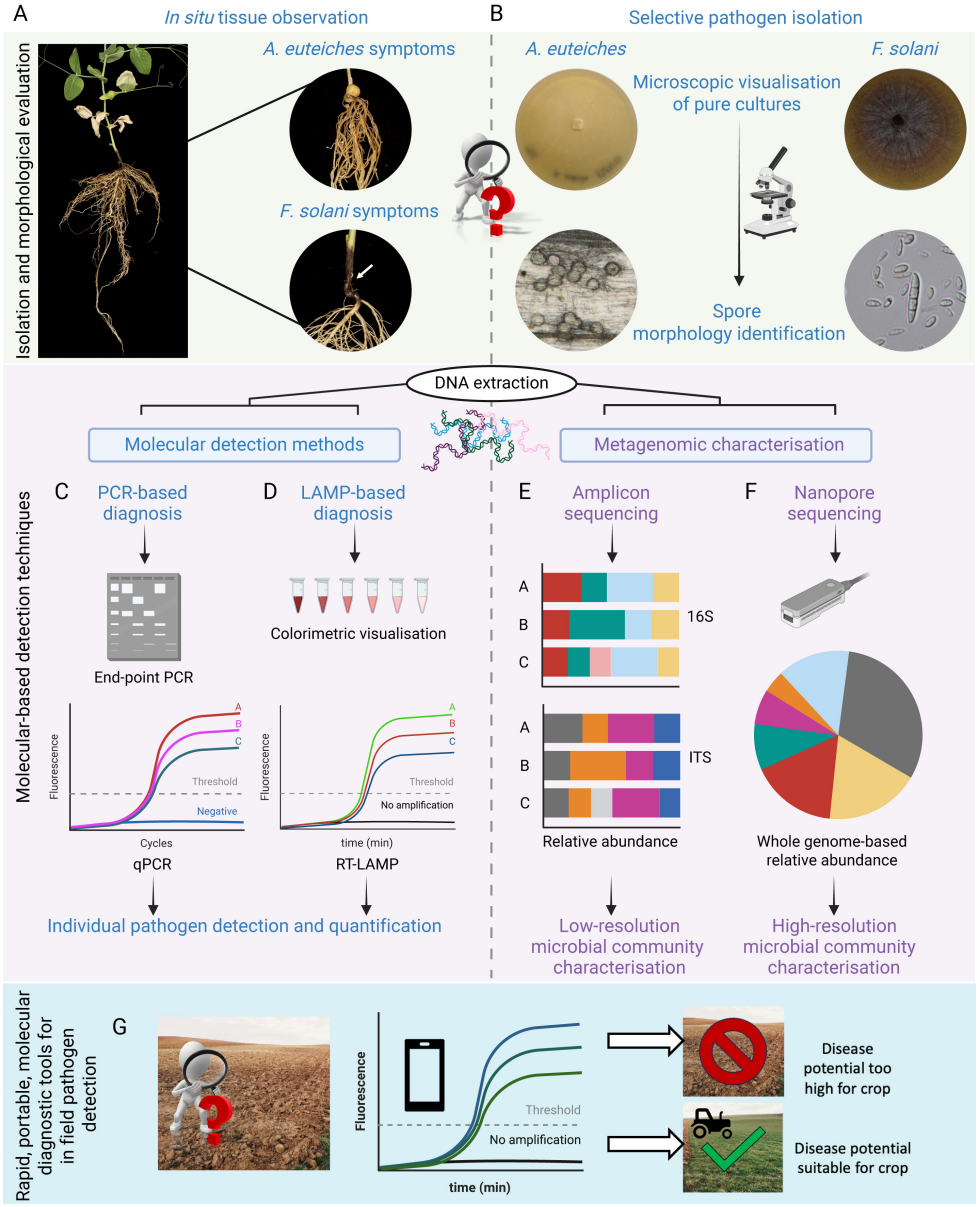
Morphological and molecular diagnosis of root rot pathogens. **(A)** Field surveys involve sampling diseased plants for visual assessment of symptoms. Severe *A. euteiches* infections display characteristic honey-brown discolouration along the entire root system. In contrast, *F. solani* infections manifest as extensive discolouration of the hypocotyl and epicotyl regions. **(B)** Infected tissue samples are used for culture-based diagnostic that allows morphological identification of pathogens. For example, *A. euteiches* oospores are spherical, 20–35 mm in diameter with a thick cell-wall which upon host recognition and germination releases bi-flagellate zoospores. *F. solani* are identified by their thick-walled macroconidia that are typically 3–5 septate with blunt and rounded apical cells and foot-shaped basal cells. Definitive pathogen identification is best achieved using molecular techniques such as **(C)** PCR or **(D)** LAMP (Loop-mediated isothermal amplification). Metagenome characterization of the associated microbiome can be achieved through either **(E)** amplicon- or **(F)** whole genome-based sequencing techniques. **(G)** Illustrates the utility of LAMP as a rapid and portable molecular diagnostic tool for identifying soil pathogens in field conditions. Real-time colorimetric LAMP results can be read via a smartphone application, allowing for quick determination of a field's disease potential. Created with Biorender.com.

Pea root rot is estimated to cause yield losses averaging 10–30%, but it can potentially lead up to entire harvest failures under optimal disease conditions (Wu L. F. et al., 2022). The disease manifests as a complex of synergistically interacting fungal and oomycete pathogens, predominantly involving *Aphanomyces euteiches, Fusarium* species (spp.), *Pythium* spp. and *Rhizoctonia solani*. These pathogens exhibit a broad host range that encompasses a variety of other crops like lentils, beans, and soybean, thereby posing a significant threat to crop yield stability. Among these, *A. euteiches, Fusarium solani* (also known as *Nectria haematococca)* and increasingly *Fusarium avenaceum* (Feng et al., 2010) have been identified as highly virulent in pea, where the first causes honey-brown water-soaked roots while the two *Fusaria* induce black lesions and rot in the hypocotyl region of the root (Figure 2A). These symptoms result in the destruction of root tissue, impeding its development, nutrient uptake, and water flow, eventually leading to a rotten root system. Under field conditions, root rot disease manifests as patches of stunted, yellowed shoots with dark brown to black roots, although this is not always readily visible, which makes diagnosis more difficult (Coyne et al., 2019). There are several factors influencing disease development, with soil type playing an important role in creating conditions conducive for pathogen proliferation (Tu, 1994). Soil moisture in particular is a key element for infection, therefore, field areas where standing water accumulates due to poor drainage will experience higher disease incidence. In addition to soil-related factors, the timing of infection and temperature can impact virulence, with plants at later vegetative developmental stages being less severely affected than seedlings.

An important factor specific to root rot disease is the co-occurrence of different complex members, with *Aphanomyces* commonly initiating primary infection, followed by opportunistic *Fusarium* spp. which often need an initial stress on the plant to facilitate infection (Wille et al., 2019; Willsey et al., 2018). The plant's transition from vegetative to reproductive phase, especially under warmer and wetter conditions, is thought to be a common trigger for *Fusarium* infection under field conditions. The co-occurring microbes have been shown to interact and mutually facilitate infection, leading to a higher disease severity compared to single inoculations (Wille et al., 2019; Willsey et al., 2018). In greenhouse trials, Willsey et al. (2018) demonstrated an increase in their assessment of root discolouration index (0–5) from 3.52 and 3.5 in single *A. euteiches* and *F. solani* inoculations, respectively, to 4.04 in combined inoculation along with reductions in root biomass. Multiplex qPCR results highlighted *A. euteiches* infection as a risk factor for exacerbated symptoms caused by secondary infection through *Fusarium* species. The study further suggested that the synergistic interactions avoid competition between pathogens due to differing colonization strategies: *Aphanomyces* leaves the vascular cylinder intact and colonizes across the whole root system, while *Fusarium* penetrates the vascular tissue and predominantly attacks the taproot near the cotyledonary attachment area. A comprehensive understanding of the mechanisms underlying these factors will form the basis of cultural control strategies. These should be coupled with the availability of cost-effective diagnostic tools, the use of good quality seeds and soil management practices that avoid soil compaction.

Furthermore, root rot pathogens, with their soil-borne and saprophytic lifestyles, often develop long-term resting structures that can survive in the soil for up to 10–15 years. This manifests as an increase in disease pressure caused by short rotation frequencies in pea cultivation (Bainard et al., 2017). It has been shown that plant health status plays an important role in shaping the soil microbiome, particularly in narrow crop rotations where pathogenic fungi may outcompete beneficial microbes, leading to decreased fungal diversity (Bainard et al., 2017). In fields where the pathogen levels have built up over the years, relying solely on climatic predictions may prove inadequate to prevent yield losses. Consequently, crop rotation with non-hosts emerges as the only effective cultural control method, demanding sufficiently long (6–8 years or even more) timeframes (Gossen et al., 2016; Williamson-Benavides and Dhingra, 2021; Hossain et al., 2012). However, such prolonged rotations hinder the efforts aimed at scaling up pea production to keep up with the burgeoning demand of the plant protein market. Hence, growers must be aware of the disease pressure in their field to effectively mitigate this issue.

## 3 Advances in field diagnostics allow informed disease avoidance for growers

In the specific case of the pea root rot complex, early detection of the pathogens involved is crucial for growers' decision making. For instance, in the UK, vining pea fields must be close to the freezing facility, no more than 150 min away. This poses a huge limitation on the fields that can be sown, and often, due to the costs and time associated with the current tests, growers act reactively and only consider assessing their crops once the disease is already established and visible. Therefore, developing a rapid, cost-effective and point-of-care detection system could greatly impact how the risk associated with this crop is managed (Bennett et al., 2012; PGRO, 2016).

For many years, field surveys have been conducted to understand the distribution and incidence of pea root rot pathogens across different regions (Hwang and Chang, 1989; Oyarzun, 1993). Plant disease diagnosis has traditionally relied on visual characterization of key signs and symptoms in the crop. Normally, the first step is to perform a visual assessment (Figure 2A) of the root rot severity based on a root discolouration scale, followed by calculating the root rot incidence per field as the percentage of symptomatic plants divided by the total number of plants sampled (Chittem et al., 2015). However, this is not always sufficient to achieve a definite diagnosis. It is often followed by more in-depth analysis carried out in a laboratory setup, involving pathogen isolation on selective culture media to evaluate traits such as spore morphology, colony color or conidiogenesis (Figure 2B) (Kumar et al., 2016; Donoso and Valenzuela, 2018).

Isolation of the pathogens involved in the pea root rot complex is routinely performed from diseased roots rather than soil (Chittem et al., 2015). This can be achieved through the method of soil baiting which involves planting a susceptible pea cultivar into a contaminated soil sample from the field and optimizing the environmental conditions to allow maximum disease manifestation. Nevertheless, culture-based techniques present some limitations for growers, (i) some distinctive characteristics of the disease can be strongly influenced by environmental conditions, leading to unreliable conclusions (ii) pathogen characterization through soil tests is time-intensive and requires specialized facilities where growers need to send their soil samples, further prolonging the time and associated costs of getting results (iii) these tests can also be expensive to run, costing upwards of £100.^1^ Therefore, utilizing molecular-based detection methods is necessary for a quicker and more definite identification of the causal agent (Figures 2C, D) (Chatterton et al., 2015; Banniza et al., 2013; Leslie and Summerell, 2008; Singh et al., 2020).

The most common molecular detection methods are based on the use of the polymerase chain reaction (PCR) to amplify and detect specific markers such as the 16S rRNA for bacteria, or the internal transcribed spacer (ITS) for fungi. PCR analysis can be undertaken for taxonomic purposes on pure cultures as a confirmation of the morphological evaluation mentioned above, or to detect the presence of pathogens responsible for the disease in a given sample without the necessity of a pre-isolation step. This approach also allows precise quantification of the pathogen by using quantitative PCR (qPCR) (Figure 2C) (Kulik et al., 2020). *A. euteiches* was first quantified by qPCR by Vandemark and co-workers in 2002 in alfalfa and, since then, this approach has been applied for the detection and quantification of *A. euteiches* and other members of the complex in pea (Vandemark et al., 2002; Zitnick-Anderson et al., 2018; Chatterton et al., 2023; Gangneux et al., 2014). In 2018, a study of the pea root rot complex defined specific primers for the quantification of *A. euteiches, F. solani, F. avenaceum* and *F. redolens*. This study not only detected the aforementioned set of organisms but assessed the interactions within members of the complex and their relation with disease severity (Willsey et al., 2018). In a recent study by Chatterton and Shimaila (2024), it was recommended to couple molecular quantification methods with the germination dynamics of pathogen spores. They observed the first detectable surge in pathogen levels 5–9 days after planting a susceptible crop in a diseased soil. This was caused by the germination of dormant oospores, leading to an exponential increase in zoospores, which are easier to quantify.

Ideally, a powerful diagnostic tool should produce a quick, cost-effective and reliable output. Recently, loop-mediated isothermal amplification assay (LAMP) has gained popularity as a point-of-care, alternative method to PCR for the detection of human, animal and plant pathogens (Figure 2D). The major advantage of LAMP diagnostics lies in its high specificity, quick readout and minimal equipment requirements since it is an isothermal reaction (Soroka et al., 2021). In contrast to PCR-based diagnostics, LAMP assays do not require a complicated laboratory setup or a pre-DNA extraction step. LAMP products can be detected either by the naked eye as a colorimetric change or by fluorometric methods. Utilizing fluorescence-based approaches, LAMP can be used for precise pathogen quantification in as little as 60 min (Figure 2G) (Tomlinson, 2008). Hence, developing and optimizing this technology for *in situ* detection of the pathogens involved in the pea root rot complex could facilitate the management and control of this disease.

## 4 Understanding root rot disease progression

Complementing diagnostics and achieving effective control of root rot necessitates the development of strategies that leverage host genetic resistance. Therefore, it is important to expand our understanding of how pathogens overcome host defenses. The challenge of tackling root rot stems from the fact that these pathogens actively and progressively weaken their host's ability to obtain nutrients—a necrotrophic strategy that distinguishes them from the more extensively studied biotrophs. Host immunity against necrotrophic root rot pathogens is often “quantitative” in nature (Jane, 2005), involving a complex network of genes and signaling pathways that together confer partial resistance through various physiological responses and pathogen inhibition. Pathogens counter these defenses by overcoming physical plant barriers and targeting multiple plant processes, making the plant-pathogen interaction a dynamic and co-evolving process.

### 4.1 Plant responses involved in root rot defense

During pathogen infection, plants employ several defense mechanisms, including maintaining a strong and dynamic root architecture, detoxifying pathogen toxins and reinforcing cell integrity to limit the pathogen root colonization. Plants respond to necrotrophic invasion through various mechanisms, such as producing antifungal compounds, occluding xylem vessels with gums, gels, or tyloses and reinforcing the cell wall through suberisation and lignification (Bani et al., 2018b). Proteomic analysis of resistant vs. susceptible pea accessions during *F. oxysporum* infection has shown that resistant plants showed increased lignin biosynthesis and depositions of cell wall appositions, called papillae, at sites of fungal penetration (Castillejo et al., 2015). A recent study also investigated the role of enzymes synthesizing phenylpropanoid compounds (which incorporate phenolics into lignin or suberin), namely guaiacol peroxidase (GPX), phenylalanine ammonia-lyase (PAL), and polyphenol oxidase (PPO), all of which showed increased expression in resistant bean accessions (Garces-Fiallos et al., 2022). Notably, the mechanism of xylem vessel occlusion helps plants maintain water and nutrient transport while blocking pathogen spread. One documented mechanism in literature is phloem anastomoses between vascular bundles, a short-term process providing alternative pathways around wounded vascular tissue (Aloni and Barnett, 1996; Aloni and Peterson, 1990). Although primarily discussed in the context of wounded stem internodes, this response demonstrates the dynamic nature of the vascular system. In addition, there is a growing interest in the role of non-coding RNA (ncRNA) in mediating plant immunity against pathogens in addition to abiotic stresses. Studies conducted in chickpea and soybean have found differential expressions of ncRNA in resistant and susceptible cultivars (Jha et al., 2021). However, this area still requires further investigation.

Besides physical defense, an important part of the plant defense reaction is the release of antimicrobial compounds. As described by Hadwiger (2008), the compounds which impede fungal colonization of plant tissues, include saponins, phytoalexins like pisatin, defensins and enzymatic degradation of fungal cell wall components using chitinase and β-glucanase. Research on non-host resistance of pea endocarp tissue to the bean pathogen *F. solani* f. sp. *phaseoli* compared to the compatible *F. solani* f. sp. *pisi* (*Fsp*) revealed these compounds were deployed more rapidly in the non-host response (Hadwiger, 2008, 2015). The genetic factors underlying the production of these compounds are often classified as pathogenesis-related (PR) genes. These genes can either encode enzymes involved in the production of antifungal compounds or proteins with direct antifungal action. A well-studied example is the phytoalexin pisatin, whose synthesis depends on the PR proteins PAL, an enzyme involved in catalyzing the first step of the phenylpropanol pathway (Hadwiger, 2008; Kawamata et al., 1992) and chalcone synthase (CHS), a key regulator of flavonoid and isoflavonoid synthesis (Hadwiger, 2008; Ichinose et al., 1992). Pisatin is a small isoflavonoid secreted by plants and taken up by fungi (Hadwiger, 2009) which causes “cytoplasmic granulation, disorganization of the cellular contents, rupture of the plasma membrane and inhibition of fungal enzymes” (Bizuneh, 2021). It is induced by fungal elicitors including oligogalacturonides (Selim et al., 2017), DNase (Klosterman et al., 2001) or chitosan (Kendra et al., 1989). Interestingly, studies investigating Ascochyta blight in pea showed that pathogens secrete both elicitors and suppressors of pisatin expression (Ichinose et al., 1992; Yamada et al., 1994), delaying the upregulation of *PAL* and *CHS*, thereby delaying the plant defense reaction. However, the key differences between compatible and non-host interactions manifest in the degree of elicitation/suppression by the fungus and the subsequent defense response, which only temporarily suppresses the compatible pathogen, eventually leading to a full infection (Hadwiger, 2008). On the other hand, PR genes directly expressing antifungal products are sometimes termed defensins. An example is *Disease-Resistance Response 230* (*DRR230*), which is highly expressed in the pea endocarp tissue during the non-host resistance response to *F. solani* f. sp. *phaseoli* (Fristensky et al., 1985; Lai et al., 2002). Defensins are diverse, low-molecular-mass cysteine-rich peptides found in mammals, fungi, insects and plants (Selitrennikoff, 2001) and classified into four groups. Their functions can range from causing morphological changes in fungi to inhibiting their growth, primarily by inducing membrane destabilization and inhibiting important cellular processes.

**Figure 3:**
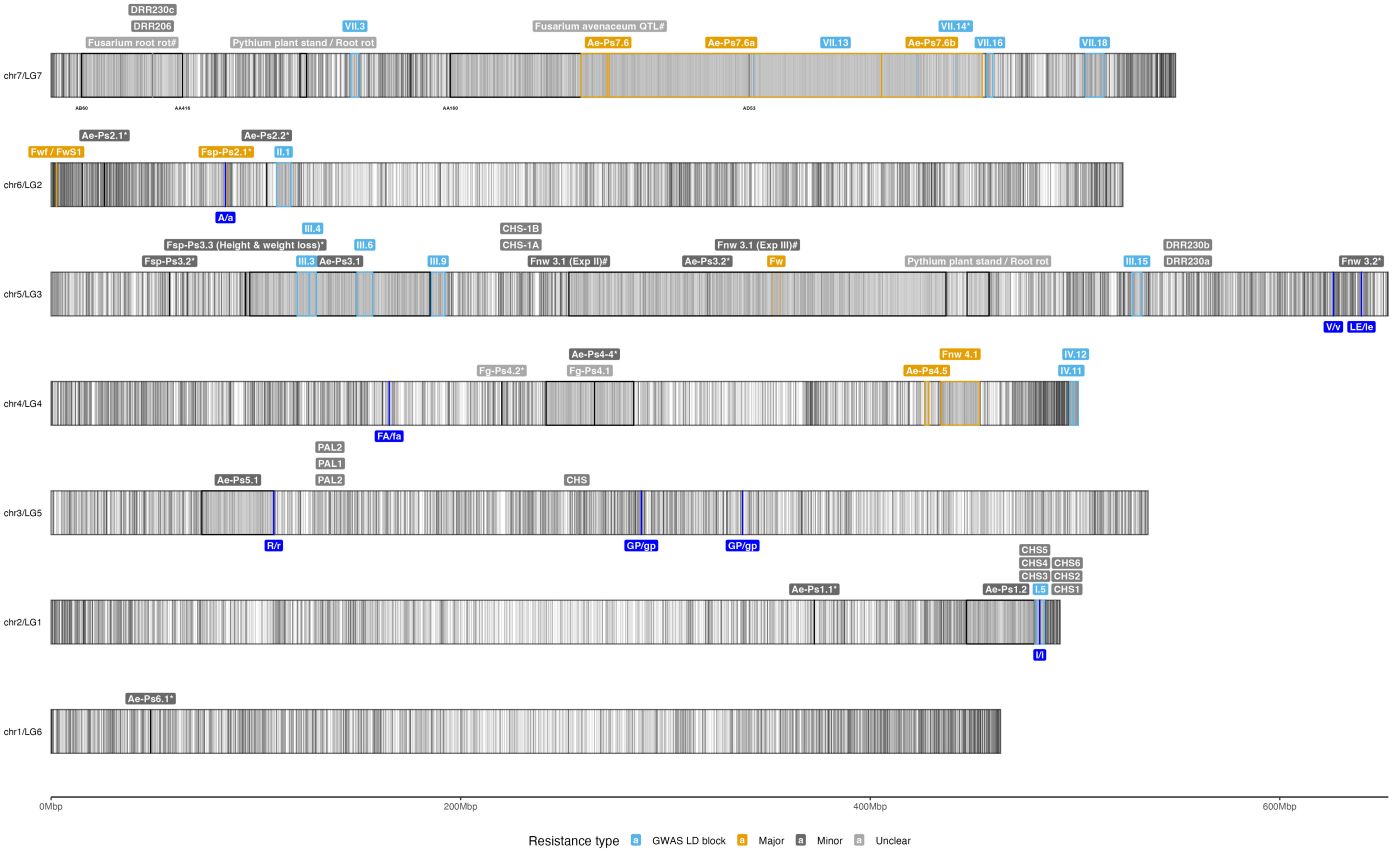
Idiogram of selected genetic resistances identified against soilborne pathogens. The figure was created using the pea Zhongwan6 genome assembly (CAAS_Psat_ZW6_1.0) (Yang et al., 2022) and visualized with the R package “ggplot2” (Team RC, 2016). The GFF3 annotation file was imported using the “rtracklayer” package and filtered for “gene” annotations. A total of 46,538 genes were plotted as vertical black lines to represent gene density. Key genetic resistance factors discussed in Section 6.1 were then plotted with labels above their respective chromosome intervals. Each interval is outlined by a colored box, indicating the type of resistance: highly consistent and significantly identified LD blocks using GWAS (light blue), major QTLs (gold), minor QTLs (dark gray), pathogenesis-related genes (red) and undefined factors (gray). An asterisk (^*^) denotes intervals that lack a second genetic marker, while a hash (#) indicates markers with ambiguous hits on the genome. Additionally, genes or candidates for Mendel's traits, as provided in Supplementary material 12 of Yang et al. (2022), are labeled dark blue beneath their chromosome positions. The markers delimiting certain intervals were also added below the chromosome to aid identification. Further details on the genetic factors are available in Supplementary Table 1.

The locations of some of these genetic factors can be visualised on the pea genome (Figure 3) and will be further discussed in section 6.1. These studies elucidate the mechanisms involved in resistance in the pea endocarp, highlighting the wide variety of defense strategies plants can employ. Despite an impressive arsenal of highly effective antimicrobial compounds, subtle differences in the timing, specificity and volume of pathogen suppression can ultimately determine the fate of the infection. It is therefore essential for a successful pathogen to overcome these defenses to establish a successful infection.

### 4.2 Factors underlying root rot pathogenicity in pea

Many *Fusarium* species exhibit a hemibiotrophic lifestyle, which starts with a brief biotrophic phase followed by a necrotrophic phase. During the necrotrophic phase, these pathogens actively weaken their host's defenses and induce cell death, employing a multitude of processes. Central to their infection strategy are cell-wall degrading enzymes, for example, *Fusarium* species are known to extensively use cellulases, pectinases, proteases and lipases to degrade major cell wall components (Perincherry et al., 2021). Additionally, these pathogens can deploy host-specific approaches, such as secreting toxins and effector proteins, to suppress plant defenses and facilitate infection and colonization. The progression of root disease in *Fusarium oxysporum* has been well studied, revealing how the degradation of the vascular parenchyma and cortical cells facilitates pathogen proliferation in susceptible pea accessions (Bani et al., 2018b). Nevertheless, our understanding of the role of toxins and other pathogenicity factors in other causal agents of pea root rot remains limited.

Despite seemingly being a simple onslaught of toxins, necrotrophic interactions involve a high degree of crosstalk and complexity compared to the well-established gene-for-gene interactions of biotrophs. For example, 746 small secreted proteins have been identified in the genome of *F. solani* f. sp. *pisi*, implicated in its adaptation to ecological niches (Coleman, 2016). A key example of this complexity is the pathogen's interaction with the antifungal phytoalexin pisatin. To establish a successful compatible interaction, the pathogen must overcome the plant's PR gene-mediated defense. Research in *N. haematococca* (*F. solani*) mating population VI (Coleman et al., 2011) has shown that this pathogen circumvents host inhibition via two mechanisms: (i) “nondegradative tolerance” involving pisatin efflux through the ATP-binding cassette (ABC) transporter NhABC1 to prevent intracellular accumulation (Hadwiger, 2008; Coleman et al., 2011) and (ii) enzymatic detoxification of pistatin through the expression of *pisatin demethylase* (*PDA*, a cytochrome P450), which converts pisatin to the less-toxic 6a-hydroxymaackiain. While both mechanisms contribute to tolerance individually, mutational studies showed that double mutants exhibit significantly impaired pathogenicity (Coleman et al., 2011). Furthermore, they proposed their deployment in a sequential manner, where the energy expenditure from the active transport of pisatin leads to the expression of the glucose-repressed *PDA1* as an additional detoxification mechanism. In *Fusarium*, the characterization of six *PDA* genes revealed that *PDA1-1* and *PDA4* are linked to high virulence in pea, with *PDA1-1* being part of the *pea pathogenicity* (*PEP*) gene cluster located on a supernumerary chromosome (Coleman, 2016). These chromosomes can be lost without affecting fungal growth and may facilitate colonization of new niches. Indeed, the acquisition of such a chromosome by a non-pathogenic isolate could lead to an opportunistic switch from saprophyte to pathogen. *PDA* genes have been used as targets for PCR-based detection of virulent *Fusarium* isolates, helping to predict high disease pressure (Etebu and Osborn, 2009).

While less studied, oomycetes also utilize strategies to weaken the host and aid colonization. For instance, *A. euteiches* employs small secreted protein effectors that target plant RNA helicases, causing nucleolar stress, thereby aiding infection (Camborde et al., 2022). Overall, necrotrophic pathogens, living in a highly competitive soil environment, employ intricate strategies to colonize their hosts, relying both on general and host-tailored factors. It is becoming increasingly clear that the distinction between a highly destructive pathogen and an opportunistic saprophyte may hinge on the presence of a few key genes (Coleman, 2016; Coleman et al., 2011). However, our detailed understanding of these molecular interactions is currently limited by the quantitative nature of the infection process and challenges in successful transformation protocols for both legumes and root rot pathogens (Larkan, 2024).

## 5 Effect of root rot on the soil microbial community

### 5.1 Characterising the microbiome dynamics during disease progression

The microbial communities inhabiting the plant rhizosphere play a crucial role in plant growth, influenced by factors like soil type, environmental conditions, plant species, genotype and root-secreted exudates (Bulgarelli et al., 2013). Pathogen invasion can disrupt the equilibrium of a healthy plant microbiome. Understanding the composition and dynamics of these microbial communities during pathogen attack is the first step in leveraging the plant microbiome in disease management. This can be achieved either by characterizing microbial markers to predict disease risk or identifying potential biocontrol agents.

To study these phenomena, amplicon-based sequencing approaches that target variable regions of specific marker genes such as 16S and ITS in the ribosomal operon in both prokaryotes and eukaryotes, are commonly used (Figure 2E) (Poretsky et al., 2014). Although this method has been applied to various pathosystems like kiwifruit—*Pseudomonas syringae* pv. *actinidiae*, chili pepper—*F. oxysporum* species complex and tomato—*R. solani* (Purahong et al., 2018; Gao et al., 2021; Kwak et al., 2018), its application to pea root rot remains limited. However, recent studies by Wille et al. (2020, 2021) have shown that diseased pea roots harbor a diverse community of fungal pathogens, including *F. oxysporum, F. solani, R. solani*, and *Didymella* spp., along with beneficial fungi like mycorrhizal species and *Clonostachys rosea*. They confirmed that the plant's health status greatly affects the fungal composition in the roots, though the impact on the bulk soil or rhizosphere is minimal. However, drawing conclusions about oomycete pathogens like *Pythium* sp. or *A. euteiches* was challenging due to underrepresentation by the amplicon primers used (Wille et al., 2020).

Resistant cultivars generally exhibited lower pathogen abundance and higher mycorrhiza presence, suggesting that *F*. *solani* and mycorrhiza abundance could serve as potential microbial markers for plant health when investigating pea root rot complex dynamics (Wille et al., 2021). While the above studies primarily focused on the impact of the disease on the eukaryotic members of the plant microbiome, it is important to also investigate the bacterial community composition, given they are the most abundant organisms in the plant microbiome. Research in Canadian commercial crop production systems in field peas revealed that bacterial root communities were most affected by the plant's health status (Hossain et al., 2021). Healthy samples showed a higher relative abundance of *Rhizobium, Olpidium* and *Morteriella* sp., but lower abundance of pathogenic *Pythium* and *Fusarium* species.

While amplicon-based studies effectively characterize microbial communities, they often lack the taxonomic precision needed for species or strain-level identification, especially when studying closely related pathogens (Edwards et al., 2015). For example, identifying *Fusarium* spp. to the species level in pea root rot complex requires analyzing multiple genes, as the widely used ITS region is often too conserved (O'Donnell et al., 2022). Metagenomic sequencing, on the other hand, offers a deeper exploration of microbial diversity and elucidation of functional attributes, revealing distinct accessory genes, like virulence factors, that can significantly influence host-pathogen interactions. This approach holds immense potential in advancing our understanding of the precise mechanisms underpinning disease pathogenesis (Levy et al., 2018). Recently, nanopore-based metagenomic sequencing has gained attention for its potential *in situ* applications (Figure 2F) (Leggett et al., 2020; Quick et al., 2016). In plant pathology, this method has been exemplified by the MARPLE (Mobile And Real-time PLant disEase) diagnostic tool (Radhakrishnan et al., 2019), developed for rapidly detecting and studying the diversity of the wheat stripe rust pathogen. Integrating metagenomic sequencing with targeted approaches promises to unravel the complexities of plant microbiome interactions, particularly in diseases involving multiple pathogens influenced by environmental factors.

Overall, the use of sequencing strategies coupled with culture-dependent approaches is indispensable to understand the complex interactions taking place within the plant rhizosphere. This knowledge enables identification and validation of key microbial groups and traits involved in pathogen suppression, ultimately informing breeding programs aimed at enhancing disease resistance and microbiome shaping.

### 5.2 Microbiome responses and the importance of root exudates

In the complex microbiome surrounding plant roots, plants must selectively combat or tolerate pathogens while simultaneously recruiting beneficial microbes. Beyond direct defense mechanisms, plants also use indirect responses that influence their susceptibility to diseases. One such strategy involves the secretion of up to 21% of their total photosynthetically fixed carbon into the soil, enriching the microbial community around the roots, a phenomenon known as the rhizosphere effect. The molecules secreted into the soil are called root exudates, a diverse and complex array of organic substances secreted by living roots into the rhizosphere (Tkacz and Poole, 2015; Lundberg et al., 2012; Huang et al., 2019; Zhalnina et al., 2018; Turner et al., 2013). These exudates include simple sugars, amino acids, carboxylic acids, secondary metabolites, mucilage and proteins (Bais et al., 2006; Compant et al., 2010; Lugtenberg and Kamilova, 2009). The composition of these exudates varies depending on several factors such as plant species/genotype, stress status, growth stage and even different sections of the root. These molecules serve multiple functions, from mediating plant-plant interactions (allelopathy or inducing herbivore resistance) to modulating plant-microbe interaction processes in the rhizosphere by actively recruiting, inhibiting or killing soil microorganisms (Lamichhane et al., 2024).

In the context of plant-pathogen interactions, the role of root exudates has been studied across several pathosystems, including root rot. For instance, pea root exudates have been shown to enhance *A. euteiches* oospore germination by ~11% compared to a water control (Shang et al., 2000). Further research on zoospores demonstrated that arabinogalactan proteins (AGPs) from pea exhibit a much stronger chemoattractant capability than those from *Brassica napus*, implicating a role in disease establishment (Cannesan et al., 2012). In contrast, root exudates from faba bean, a more tolerant host toward *A. euteiches*, had a negative effect on zoospore chemotaxis, likely due to the presence of furanoacetylenic compounds (Laloum et al., 2021). However, root exudates can also have an inhibitory effect on root rot progression as reported for both *A. euteiches* and *Fusarium* spp. For example, the tips of pea roots are disease-free zones, likely due to the production of the phytoalexin pisatin by root border cells (Pueppke and VanEtten, 1976; Bani et al., 2018b; Cannesan et al., 2011). Moreover, in response to pathogen attack, plants can release root exudates that recruit beneficial rhizosphere microorganisms, a process known as the “cry for help” response. This response assembles a consortium of beneficial microbes capable of inhibiting pathogen colonization (Rolfe et al., 2019). This phenomenon has been extensively studied in systems such as *Arabidopsis*-*Hyaloperonospora arabidopsidis* (Berendsen et al., 2018), sugar beet-*R. solani* (Mendes et al., 2011) and common bean. For example, in common bean, specific bacterial taxa, such as *Pseudomonadaceae, Bacillaceae, Solibacteraceae*, and *Cytophagaceae*, were found to correlate with resistance to *F. oxysporum*. Furthermore, the resistant cultivar exhibited a more complex and highly interconnected bacterial community structure (Mendes et al., 2018). Other examples are the recruitment of beneficial growth-promoting microbes like *Rhizobium*, which have demonstrated the ability to alleviate disease severity (Bani et al., 2018b; Kalantari et al., 2018; Ranjbar Sistani et al., 2017; Makarova et al., 2016; Short and Lacy, 1976). By understanding these multifaceted strategies, we can develop effective approaches to combat pea root rot pathogens.

## 6 Exploiting host genetic resistance to combat root rot pathogens

As root rot is challenging to manage with chemical and cultural methods, durable genetic resistance through the identification of QTL that distinguish partially resistant from susceptible accessions is a key to breeding for resistance. However, we currently lack a functional understanding of these responses at the gene level, hindering our ability to fine-tune resistance and further understand the complexities of these pathogens. Given that root rot resistance involves multiple small-effect genes, phenotyping large, genetically diverse and densely genotyped pea mapping populations against various pathogens is necessary. This approach will increase the statistical power needed to uncover genetic resistance, which can eventually be pyramided in a cultivar to provide multi-pathogen resistance. To achieve this, novel sources of genetic resistance within the *Pisum* species must be identified and utilized.

*P. sativum*, one of the oldest domesticated crops originating from the Middle East, includes the domesticated *P. sativum* ssp. *sativum* grouped together with the wild subspecies *P. sativum* ssp. *elatius*, distinct from *P. fulvum* and *P. abyssinicum*. Evidence suggests that domesticated pea likely originated from *P. sativum* ssp. *elatius*, while *P. abyssinicum* has undergone an independent domestication process (Coyne et al., 2020; Smýkal et al., 2012; Ambrose, 1995; Timo et al., 2022; Kreplak et al., 2019; Trněný et al., 2018). For breeding, the *P. sativum*. ssp. *elatius* and cultivated pea form the primary gene pool, while *P. fulvum*, despite reduced fertility (Bogdanova et al., 2014) can intercross with this pool and is part of the secondary gene pool. *P. abyssinicum*, due to its distinct diversity, is sometimes also included in this gene pool. The broad genetic diversity and co-evolution of these wild relatives with various pathogens emphasizes their value in germplasm collections. Most of *Pisum's* genetic diversity lies outside of the cultivated accessions (Jing et al., 2010), representing a reservoir of untapped genetic resistance, as demonstrated by *P. fulvum's* resistance to the pea bruchid weevil (Hardie et al., 1995).

**Figure 4:**
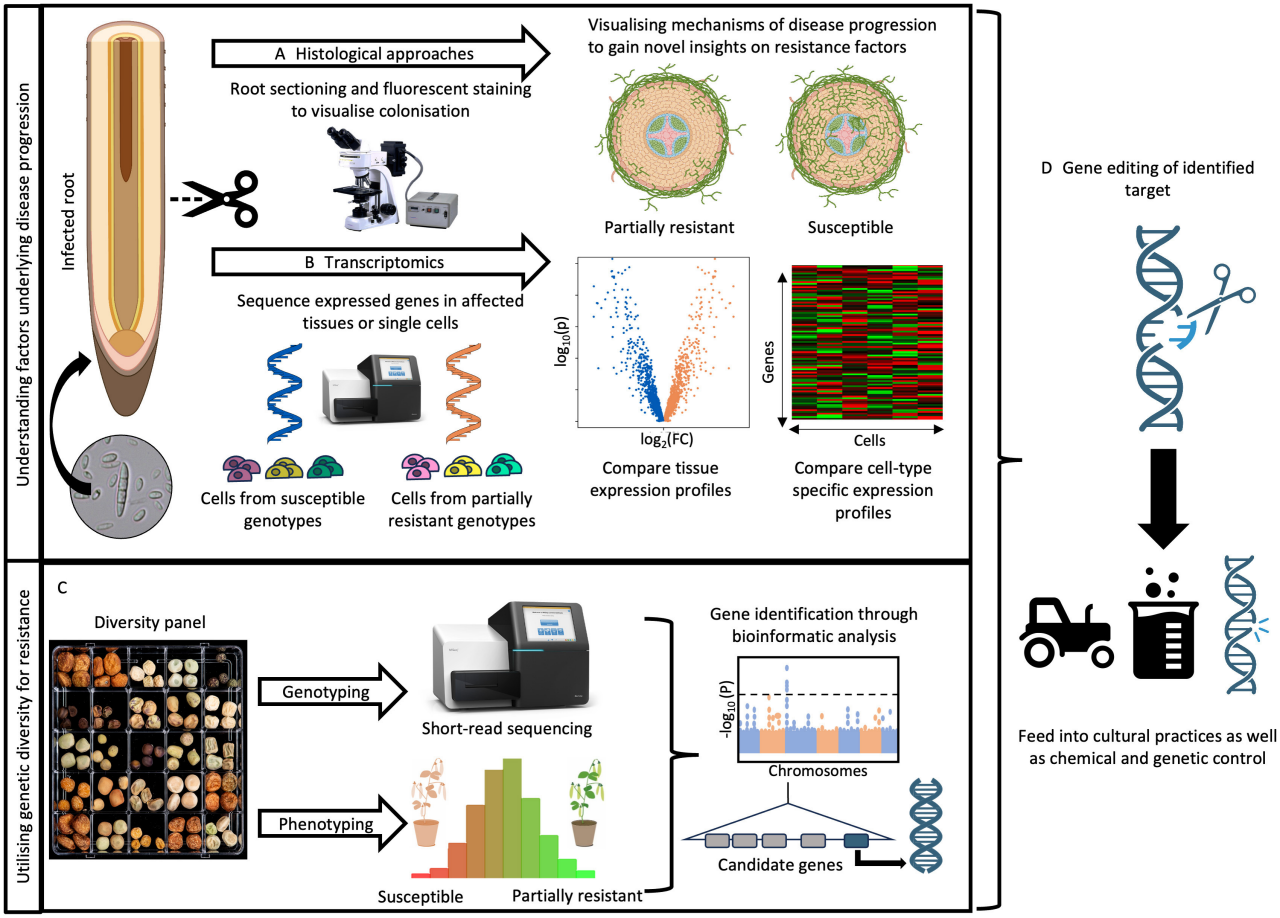
Identification and characterization of host genetic factors underlying root rot resistance. **(A)** The progression of root rot pathogens is impeded by the physical reinforcement of cells through suberisation, lignification and papillae formation. Histological studies, such as those using wheat germ agglutinin fluorophores, can visualize fungal colonization in susceptible and partially resistant genotypes to help identify root compartments crucial for halting pathogen progress and preventing widespread colonization of the cortex and vasculature. Root section cartoons created with Biorender.com. **(B)** Once these critical compartments of the root are identified, techniques like microdissection or single-cell RNA sequencing would enable comparison of cell-type-specific gene expression profiles, characterizing the crucial genetic factors that halt pathogen colonization in roots. **(C)** Genetic resistance to root rot pathogens can be identified by implementing association mapping approaches on a genotyped *Pisum* diversity panel or using biparental populations. The linked genetic loci can be further fine mapped to identify candidate genes. **(D)** Together, these insights can be utilized to design constructs targeting either resistance or susceptibility factors using CRISPR/Cas9 technology, thereby supplementing control methods.

To capitalize on this inherited diversity, comprehensive genetic and phenotypic characterization of *Pisum* germplasm collections is necessary. With falling sequencing costs and advances in computational power, whole genome sequencing (WGS) of three *Pisum* collections, encompassing a wide range of wild, landrace and cultivar accessions, has been completed (Yang et al., 2022; Feng et al., 2024; Liu et al., 2024). Moreover, various community efforts have also developed rapid, large-scale pathogen screening methods to complement genetic resources (Grünwald et al., 2003; Desgroux et al., 2018; Infantino et al., 2006; Kraft et al., 1994; Atkinson et al., 2019). To maximize the utility of these resources, efficient phenotype to genotype mapping methods, including either SNP-based or *k-mer*-based genome wide association mapping (GWAS) (Figure 4C), have enabled the identification of resistance genes in pea and other crops (Desgroux et al., 2018; Reeves et al., 2012; Desgroux et al., 2016; Leprévost et al., 2023).

### 6.1 Identified host genetic resistance against root rot pathogens

Extensive research using pea's genetic diversity has identified and mapped quantitative trait loci for resistance against various root rot pathogens through GWAS or linkage mapping approaches (Wille et al., 2019; Rubiales et al., 2023; Wohor et al., 2022; Rubiales et al., 2015; Wu et al., 2018; Rubiales et al., 2019). While the primary focus has been on dominant members of the root rot complex, particularly *A. euteiches* and *Fusarium* species, other rhizosphere pathogens have also been studied. This review provides a comprehensive overview of co-occurring root rot pathogens, offering insights into a holistic approach to managing root rot. Key genetic factors involved in resistance are discussed and mapped onto an ideogram of the Zhongwan 6 (ZW6) reference genome (Yang et al., 2022) in Figure 3 and detailed in Supplementary Table 1.

#### 6.1.1 *Fusarium solani* f. sp. *pisi (Nectria haematococca)*

First described in 1918 (Bisby, 1918), *Fsp* is a prevalent and common cause of root rot worldwide. *Fsp* infection, or *Fusarium* root rot, is characterized by brown/black lesions on the hypocotyl region accompanied by red vascular colouration, extensive root decay and wilting. Infection begins when dormant spores perceive root exudates and germinate (Smith, 2007). Fungal spores attach to hosts using spore tip mucilage, which can be induced by treatment with plant extracts (Coleman, 2016). Once the fungus comes into contact with the host plant surface, hyphae will grow and proliferate on its surface until they find an entry point like stomata, wounds or thin barriers (Wohor et al., 2022; Smith, 2007). Upon penetration, the fungus spreads intracellularly into the upper portion of the taproot through epidermal and parenchyma cells, causing destruction of the middle lamella and cell death, eventually colonizing the xylem (Coleman, 2016). Disease progression is marked by coalescing lesions, vascular discolouration and growth cessation, leading to early senescence or plant death in severe cases. The lifecycle is completed with the production of the thick-walled chlamydospores that facilitate subsequent infections (Wohor et al., 2022; Smith, 2007).

Screening of various cultivars and germplasm collections has identified several QTLs conferring partial resistance to *Fsp* in pea (Coyne and Pilet-Nayel, 2008; Kishore et al., 2015; Williamson-Benavides et al., 2020). Wildtype accessions with pigmented flowers and seed coats, such as PI 125673 and PI 175226 (Bodah et al., 2016), exhibit stronger resistance, possibly linked to the *A* locus on chr6/LGII (Hellens et al., 2010). Genetic mapping identified a QTL on chr6/LGII overlapping the *A* locus, along with additional QTL on chr4/LGIV and chr7/LGVII (Weeden and Porter, 2007). Evidence suggests that the chr4/LGIV QTL confers resistance only in the presence of the chr6/LGII QTL, indicating a role in the anthocyanin/polyphenol/flavonoid pathway. Furthermore, a field study using a recombinant inbred line (RIL) population identified a major QTL on chr7/LGVII (Feng et al., 2011). Other studies pinpointed (Coyne et al., 2015) and confirmed (Coyne et al., 2019) the major QTL Fsp-Ps 2.1 on chr6/LGII in a 1.2 cM confidence interval, explaining up to 53.4% of phenotypic variation, along with several minor effect QTL on chr4/LGIV, chr1/LGVI, chr7/LGVII and chr5/LGIII. The underlying genes linked to these QTL are not yet fully characterized but are expected to involve transcription factors, stress-associated phytohormones, PR proteins, and the pea phytoalexin pisatin. Recent RNA-seq analysis of partially resistant and susceptible accessions identified additional QTL and revealed the involvement of DRR230 and genes related to sugar transport, receptor-mediated endo- and exocytosis, cell death, and anthocyanin synthesis pathways (Williamson-Benavides et al., 2020).

#### 6.1.2 *Aphanomyces euteiches*

*A. euteiches* is a key oomycete and most widely studied pathogen causing root rot in pea. Infection starts when zoospores are released from dormant oospores upon perceiving host exudates leading to root colonization. The affected plants become stunted, develop honey-brown discolouration across the root system, and may eventually wilt, causing yield losses from 10% to 86% in severe cases (Wohor et al., 2022; Wu et al., 2018; Benavides, 2020). Significant efforts have been made to identify resistance in the pea germplasm. For example, Conner et al. (2013) identified the accession “00-2067” showing high levels of disease tolerance while maintaining vigor and yield under high disease pressure, making it a strong candidate for resistance breeding. A recent bulk segregant RNA-seq study using the resistant accession “00-2067” identified genes linked to root development, immune responses, and signaling pathways (Wu L. et al., 2022).

Several studies have mapped major QTL conferring partial resistance to *A. euteiches*, including *Ae-Ps4.5* on chr4/LGIV and *Ae-Ps7.6* on chr7/LGVII, as well as a number of minor QTL on various chromosomes (Davis et al., 1995; Pilet-Nayel et al., 2002; Pilet-Nayel, et al., 2005; Hamon et al., 2011). These major QTL have been further validated using near-isogenic lines (NILs), along with minor QTL *Ae-Ps2.2* and *Ae-Ps5.1*, using two reference strains of *A. euteiches* (Lavaud et al., 2015). In recent developments, the major QTL *Ae-Ps4.5* has been fine mapped to a 3.06-Mb interval underlying 50 annotated genes in the Caméor v1a reference genome (Lavaud et al., 2024). A comparative GWAS on root system architecture (RSA) and partial resistance to *Aphanomyces* in 266 pea accessions revealed that the major QTL *Ae-Ps7.6* is associated with root system architecture. It suggested that increased root area enhances resistance, complementing the observation that longer roots with higher numbers of lateral roots are associated with stronger resistance by allowing the plant to enhance the uptake of resources and creating a less favorable environment for the pathogen (Desgroux et al., 2018). This observation has also been reported for *Fusarium* root rot in bean (Román-Avilés et al., 2004) and pea (Kraft and Boge, 2001).

In addition, a major QTL has been identified for *A. euteiches* root responses in *Medicago truncatula*, a close relative of *P. sativum* (Djébali et al., 2009). A local score approach which considered “the surrounding signal due to linkage disequilibrium” in GWAS has improved resolution to detect small-effect loci which identified four novel QTL in *M. truncatula* related to pathogen effector recognition and plant proteasomes (Lavaud et al., 2024). An important factor during resistance screening is considering the population structure of *A. euteiches*. High genetic diversity has been observed in USA populations (Grünwald and Hoheisel, 2006; Malvick and Percich, 1998), while low to moderate diversity has been reported in pea-growing regions of France (Wu L. et al., 2022; Kälin et al., 2022; Quillévéré-Hamard et al., 2018; Wicker and Rouxel, 2001). This variability suggests that breeders must remain vigilant and use practical soil-screening methods to monitor pathogen populations and respond to changes.

#### 6.1.3 Seed and seedling rots caused by *Rhizoctonia* spp. and *Pythium* spp.

While *A. euteiches* and *F. solani* are the primary agents of root rot, seed and seedling rots, often termed “pre-emergence and post-emergence damping-off”, are typically caused by minor pathogens like *Rhizoctonia* and *Pythium* spp., specifically *Rhizoctonia solani* and *Pythium ultimum*.

*P. ultimum*, an oomycete, infects peas and a broad range of other hosts during emergence via encysting and invading zoospores, leading to germination issues and watery-brown root discolouration (Wohor et al., 2022; Schroeder et al., 2013). Similar to *A. euteiches*, the disease is promoted by waterlogged conditions and the pathogen survives as oospores in the soil. Thus, cultural practices such as avoiding soil compaction, increasing soil organic matter content, crop rotation and using uncontaminated seeds are important to avoid strong disease symptoms (Wu et al., 2020). Additionally, using fresher and larger seeds could reduce disease severity (Kraft et al., 1994). It has been reported that genotypes with the *A* locus were more resistant due to the presence of the anthocyanin delphinidin in their seed coat. Although some resistant lines have been identified (Kraft and Roberts, 1970), due to the nonspecific nature of symptoms and overlapping species (Wohor et al., 2022; Schroeder et al., 2013), we have limited understanding of resistance in peas. Nevertheless, Klepadlo et al. (2019) utilized a RIL population in soybean to identify QTL on chromosome 6 and 8, explaining 7.5–13.5% and 6.3–16.8% of phenotypic variation, respectively and identified potential candidate genes including Ring/Zinc-finger proteins, MYB family transcription factors, leucine-rich repeat-containing resistance protein kinases and receptor-like proteins. The orthologs of these regions (Yates et al., 2022) can be used to obtain putative regions in the pea genome which map to chr5/LGIII and chr7/LGVII.

*R. solani* is a major cause of *Rhizoctonia* root rot, primarily infecting the epi-/hypocotyl or seed, leading to damping-off and stunted plant growth. The fungus is most aggressive under warm, moist conditions and grows best on well-aerated soil surfaces. Infection begins when vegetative mycelia and sclerotia detect and grow toward roots or seeds, penetrating through soft spots or wounds. The resulting tissue destruction and sclerotia formation cause soggy lesions, mainly in the collar region (Kraft and Pfleger, 2001; Kraft et al., 1994; Wohor et al., 2022; Sharma-Poudyal et al., 2015). There is limited knowledge on resistance to *Rhizoctonia* root rot but it has been associated with epicotyl thickness and seedling age, where older seedlings are less susceptible (Wohor et al., 2022). Although genetic factors for resistance in pea are not well understood, Wang and Fristensky (2001) have demonstrated that the constitutive overexpression of the pea defense gene *DRR206* in canola confers resistance to *R. solani* and other fungal species (Seneviratne et al., 2015). They suggested that *DRR206* may be involved in lignan synthesis which strengthens cell walls or lignifies pathogen structures. *DRR206* has been implicated in the early non-host response (Culley et al., 1995) and is associated with the synthesis of pinoresinol monoglucoside, a compound involved in phytoalexin responses (Seneviratne et al., 2015).

#### 6.1.4 Further *Fusarium* sp. involved in root rot

Among *Fusarium* species, *F. oxysporum* is a significant pathogen with a very broad host range, notably causing *Fusarium* wilt in pea in addition to being a minor member of the pea root rot complex. The pathogen invades roots, remaining asymptomatic until it colonizes the vascular tissue, leading to wilting and necrosis. Four races of *F. oxysporum* f. sp. *pisi* (*Fop*) have been described, with races 1 and 2 occurring worldwide, while races 5 and 6 are specific to America (Bani et al., 2018b; Sampaio, 2020). Resistance to race 1, 5 and 6 is conferred through a single dominant gene (McClendon et al., 2002), while resistance to race 2 is quantitative in nature (Bani et al., 2018b; Mc Phee et al., 2012). *Fop* race 1 resistance was first described in 1924 (Wade, 1929) and has since been deployed into pea varieties (Wohor et al., 2022). Resistance is mapped to chr5/LGIII for race 1 (McClendon et al., 2002; Grajal-Martin and Muehlbauer, 2002; Jain et al., 2015) and chr6/LGII for race 5 (Coyne et al., 2000) and more recently, Deng et al. (2024) refined this QTL to a 91.4 kb interval with a candidate gene pinned down to *Psat6g003960* characterized as having an NB-ARC domain. Resistance to race 2, however, is quantitative and mapped to a major QTL on chr4/LGIV and two minor QTL on chr5/LGIII (Mc Phee et al., 2012). Moreover, histochemical characterization of *Fop* race 2 revealed multiple physical and chemical barriers, primarily involving papilla formation, cell wall strengthening and accumulation of (poly)phenolics and carbohydrates, which impede fungal proliferation outside and inside the vasculature (Bani et al., 2018a).

*F. avenaceum* is increasingly recognized as a devastating root rot pathogen in the *Fusarium* genus, particularly in North America (Wu L. F. et al., 2022; Wille et al., 2020). Indeed, *F. avenaceum* has been found to be the most prevalent *Fusarium* species isolated in field surveys in North Dakota between 2004–2009 (Chittem et al., 2015; Fernandez et al., 2008). While causing similar symptoms to *F. solani*, it can be distinguished morphologically through the stoutness of the macroconidia and through ITS sequences (Feng et al., 2010). A study Awodele et al. (2024) found that pea varieties with pigmented flowers and seeds showed significantly lower root rot severity (8.0% to 43.0%) compared to white-flowered varieties (89.7%−95.0%) (Awodele et al., 2024). Genetic resistance to *F. avenaceum* is limited, however, resistance against F. avenaceum was identified and mapped to chr7/LG7 (Feng et al., 2011; Li et al., 2012) which explained QTL on chr7/LG7 which explained 21.7% of the phenotypic variance. Interval QTL mapping analysis on markers that associated with disease severity (AA160, AD53, AA416 and AB60; all of which also showed significant association) located the interval around the distal end of the chromosome. Interestingly, two of those markers (AA416 and AB60) indeed map to the distal part of the chromosome in the ZW6 assembly and overlap with the locations of *DRR206* and *DRR230c*. Furthermore, despite the region spanning the markers AA160 and AD53 not being in the same area of the chromosome, they overlap with the mapping of the major *A. euteiches* QTL *Ae-Ps7.6*. These connections between different *Fusarium* ssp., pigmentation, non-host resistance and *A. euteiches* surely warrant further investigation.

The *F. graminearum* species complex, known for causing Fusarium head blight in cereals and producing mycotoxins, also impacts pea through root rot (Wu L. F. et al., 2022). A mapping study utilizing a RIL population identified 11 QTL associated with vigor, plant height and root rot severity (Wu L. F. et al., 2022). Two stable QTL relating to root rot severity were identified on chr4/LGIV (*Fg-Ps4.1* and *Fg-Ps4.2*), which interestingly overlaps with a minor QTL for *Aphanomyces* resistance *Ae-Ps4-4* when mapped onto the ZW6 assembly. Among the 74 candidate genes underlying the QTL region, the authors identified 15 genes related to plant defense.

### 6.2 Utilizing mutant populations to identify genetic resistance

Another approach which does not rely solely on natural diversity but instead utilizes artificially introduced diversity, is the creation of mutant populations. Several mutant populations have been developed in pea, such as the Cameor TILLING population at INRAe, France (Coyne et al., 2020) and the fast-neutron mutagenized deletion population at the John Innes Center (Domoney et al., 2013). Screening these mutant populations with root rot pathogens could identify new genetic loci or allelic variations, or aid in the functional validation of identified candidate genes by demonstrating loss-of-function phenotypes (Guo et al., 2019).

### 6.3 Translating genetic resistance to field conditions

Translating genetic resistance identified under controlled environments (like laboratories and greenhouses) to field conditions involves addressing several challenges, including environmental variability, pathogen diversity, and the complexity of resistance traits. By combining modern breeding techniques, like genomic selection and CRISPR gene editing, with a deep understanding of plant-pathogen interactions, it is possible to develop crop varieties that are not only resistant to root rot but also capable of maintaining high yields under disease pressure.

Unlike foliar diseases, root rot primarily affects the root system, posing minimal risk of yield contamination through colonization of tolerant hosts. Additionally, vigorous plants that can outgrow the damage and maintain performance, exert a substantially lower selection pressure on the host-pathogen interaction, offering a more sustainable control method. This is highlighted by a previously mentioned GWAS associating *Aphanomyces* resistance with stronger root system architecture (Desgroux et al., 2018). It has therefore been suggested to consider genetic control not merely in terms of (partial) resistance, meaning a trait leading to the reduction of disease intensity, but in terms of “field tolerance”, i.e., maintaining yield despite appearance of symptoms (Conner et al., 2013; Hance et al., 2004; You et al., 2017; Mussel, 1980).

For effective field deployment, it is also crucial to consider the underlying mechanisms of naturally occurring resistance. For instance, a gene expression analysis study conducted on *Fsp* responsive genes (Williamson-Benavides et al., 2020) showed that susceptible genotypes overexpress a broad range of defense-related genes, whereas tolerant genotypes exhibit a more targeted response. This precise response conserved energy, allowing tolerant plants to return more easily to a basal metabolic state, thereby enhancing their ability to maintain yields during complex pathogen interactions. Despite being more sustainable than monogenic resistance, tolerance and partial resistance in pea are polygenic traits, making their introgression into cultivars a laborious task requiring gene pyramiding. Nevertheless, modern genomics-enabled breeding approaches offers promising solutions to achieve this goal (Parihar et al., 2022).

## 7 Exploiting novel techniques to characterize resistance

### 7.1 Advances in genetic engineering for root disease resistance in pea

The availability of vast genetic resources offers opportunities to further characterize resistance traits at the gene-level and identify targets for genetic engineering. A common method for studying root traits is the transient expression of genes in root tissues using *Agrobacterium rhizogenes* (*Rhizobium rhizogenes*), which generates transgenic hairy roots (Srivastava et al., 2018). This technique has been used in plant-pathogen interaction studies, such as in sugar beet against *F. oxysporum*. Here, the expression of polygalacturonase-inhibiting proteins (PGIPs) inhibited the fungal enzymes that break down cell walls during early infection (Li and Smigocki, 2019). Furthermore, overexpression of the common bean *PvPOX1* gene in transgenic roots increased resistance to *F. oxysporum* f. sp. *phaseoli* in the susceptible BRB130 genotype via oxidative burst, the induction of hypersensitive responses, the expression of PR genes and significantly increasing hydrogen peroxide (H_2_O_2_) accumulation (Xue et al., 2017). Hairy roots have also been used to study fungal pathogenicity factors in the pea-*N. haematococca* (*Fsp*) interaction. In this study, roots expressing sense or antisense cDNA of enzymes involved in the synthesis of pisatin, a plant defense compound, showed increased susceptibility when pisatin production was impeded, demonstrating the importance of this compound in disease resistance (Wu and VanEtten, 2004). This technique allows testing multiple candidate genes within a relatively short amount of time, but is restricted to transgenic roots, thereby not modifying the germline for heritable and stable transformation into subsequent generations.

*Agrobacterium tumefaciens*, on the other hand is utilized for functional validation in genetically stable whole-plant transgenics. One example of how this has recently been achieved in plant root diseases is shown by Wang et al. (2023) in clubroot (*Plasmodiophora brassicae*) on *Arabidopsis*. After identifying a major dominant locus underlying nine candidate genes, the authors transformed the individual genes into the susceptible Col-0. This revealed that only one of the genes *C6* (later named *WeiTsing*) conferred strong resistance and is expressed exclusively in the root pericycle to protect the stele and codes for a cation-selective channel permeable to Ca^2+^. Conversely, CRISPR-Cas9 mediated mutation revealed two mutant alleles abolishing resistance. Gene editing using the CRISPR-Cas9 system is a valuable tool for functional validation by knockout mutations. Natural allelic variation of causal genes can guide base-editing for gain-of-function mutations (Figure 4D). However, due to the lack of high-throughput genetic transformation systems owing to recalcitrance to *A. tumefaciens*, functional validation and disease resistance engineering has been impeded in pea and other legumes (Grant and Cooper, 2003; Nguyen et al., 2016). Despite this, pea has now entered the genome editing era. CRISPR-Cas9 mediated gene knockout has recently been demonstrated in pea for *phytoene desaturase* gene (Li et al., 2023) and applied to improve flavor by knocking out lipoxygenase (LOX) enzymes (Bhowmik et al., 2023) and creating saponin-free pea seeds through editing of *PsBAS1* (Hodgins et al., 2024). Moreover, hairy root transformation can also be combined with CRISPR-Cas9 for applications in root disease research (Kiryushkin et al., 2021; Bhowmik et al., 2021; Alamillo et al., 2023).

### 7.2 Histological assays to understand root disease establishment

Histological methods can be used to microscopically track pathogen disease progression within the host, offering insights to identify critical timepoints during the early stages of infection—an area not widely explored for root rot pathogens (Figure 4A). A study on *Medicago truncatula's* response to *A. euteiches* infection compared resistant (A17) and susceptible (F83005.5) accessions to investigate disease progression across different root sections (Djébali et al., 2009). The study found that resistant plants produced significantly more secondary roots and exhibited a slower disease progression compared to susceptible plants which produced yellow-gray discolorations by 3 days post inoculation, progressing to macerated, soft brown tissues and eventually cotyledon yellowing and wilting between 15 and 21 days. Using a wheat germ agglutinin (WGA)-fluorescein isothiocyanate (FITC) conjugate to visualize oomycete hyphae and oospores using epifluorescence microscopy, the study showed that the stele of A17 remains pathogen free while being colonized in susceptible responses. Susceptible plants showed colonized cortical cells by six days post inoculation, with vascular cells fully colonized after 15 days. The resistant A17 plants exhibited strong blue autofluorescence, correlating with the accumulation of phenolic compounds in cortical cells. Furthermore, there were striking differences in the cell reinforcement around the vascular cylinder and increased cell divisions in the pericycle of resistant plants. By utilizing phloroglucinol (Wiesner reagent) the authors were able to visualize the accumulation of lignin-like compounds by 3 days post inoculation and forming a barrier surrounding the stele after 6 days for the inoculated resistant plant, while susceptible and control plants only displayed these in the cell walls of xylem vessels. These findings suggest that partial resistance involves the accumulation of phenolic compounds and reinforcement of the stele. Gaining such knowledge in pea will complement studies investigating host gene expression during infection to further refine gene candidates that support genetic resistance.

### 7.3 Transcriptomics in unraveling root disease mechanisms

A key finding from recent histological studies is that different root regions interact distinctly during pathogen invasion, and protecting the root stele is crucial in preventing systemic infection. This suggests that different cell types have unique expression profiles, which might be overlooked in whole-tissue transcriptomic studies. To address this, modern techniques like single-cell transcriptomics have been developed (Figure 4B).

Single-cell transcriptomics has been primarily used to study leaf developmental processes (Lopez-Anido et al., 2021; Kim et al., 2021; Liu et al., 2020) with some studies of cell type identity in *Arabidopsis thaliana* roots (Dorrity et al., 2021; Shahan et al., 2022) and root apical stem meristematic tissue in rice (Zhang et al., 2021). Recently, it has been applied to study plant-pathogen interactions. For example, Tang et al. (2023) used droplet-based single-cell RNA sequencing to create a leaf cell atlas of 95,040 cells during fungal infection by the hemibiotrophic *Colletotrichum higginsianum*. The study highlighted cell-type specific expression and revealed that intracellular immune receptors were enriched in vascular cells. They also utilized trajectory inference (Saelens et al., 2019; Deconinck et al., 2021) and live-cell imaging to associate specific cells at infection sites with transcriptional reprogramming of abscisic acid signaling in guard cells. Particularly, epidermally expressed *MYB122*, involved in glucosinolate biosynthesis, was identified as a contributor to disease resistance, as mutants displayed hyper susceptibility.

Single-cell transcriptomic studies in root diseases are less common. One example is Cao et al. (2023) who investigated stalk rot (*Fusarium verticillioides*) in maize roots. They analyzed 29,217 root tip cells from a susceptible and a resistant inbred line, identifying 12 pathogen-responsive regulatory modules. A machine-learning approach predicted resistance-associated genes, specifically highlighting differential expression of genes involved in the phenylpropanoid pathway within the cortex, stele and vasculature. Subsequent virus-induced gene silencing of the genes in this pathway (*ZmPAL6, ZmCOMT* and *ZmCCoAOMT2*) led to significantly increased disease severity and reduced lignin content, indicating weak lignification of cell walls of hypodermal cells and vascular tissues. Additionally, silencing *ZmPAL6*, a key enzyme in the synthesis of salicylic acid, increased disease severity from other causal agents of stalk rot *F*. *proliferatum* and *Pythium aristosporum*.

Laser capture microdissection (LCM) is another method that allows spatial transcriptomic investigation by isolating selected cell populations, even at the single-cell level (Balestrini et al., 2009). LCM has been used to study Arbuscular Mycorrhizal (Hogekamp et al., 2011) and *Rhizobium* (Schnabel et al., 2023) symbiosis in *Medicago truncatula* and pea (Kusakin et al., 2021). In plant-fungal pathogen interactions, LCM was first demonstrated in 2006 to study maize-anthracnose stalk rot interactions which revealed fungal DEG (Tang et al., 2006). It has also been applied to investigate *Arabidopsis* roots inoculated with the clubroot causing protist *P. brassicae* (Schuller et al., 2014). The study isolated cells from specific developmental stages during disease progression, confirming the role of genes involved in auxin and cytokinin metabolism and signaling and the involvement of increased brassinosteroid (BR) synthesis in gall formation, which could be reduced by BR inhibitors.

All these cutting-edge developments are emerging as valuable tools to dissect host responses in various plant tissues that the pathogen needs to overcome. Further unraveling the complexity of different host compartments providing variable environments for the pathogen will enhance our understanding of host-pathogen interactions.

## 8 Conclusion

Numerous positive developments highlight the increasing availability of functional and genetic resources enabling the elucidation of molecular factors underlying disease resistance against root rot to a degree which has thus far eluded us. However, this resistance is not simply the result of single host-pathogen interactions, but rather a complex and dynamic interplay involving the host, its microbiome, multiple co-occurring pathogens that amplify each other's virulence and environmental conditions affecting disease severity. As the manifestation of these responses can vary considerably, it is important to further dissect factors that differentiate a susceptible from a partially resistant plant. Instead of eliminating the pathogen, the focus is on “field tolerance” in pea accessions capable of maintaining substantial yields despite high pathogen pressure in a field. Allowing restricted proliferation while still curbing optimal conditions will provide a more durable form of resistance compared to actively engaging with the pathogen. Coupled with highly specific portable diagnostic methods determining the disease potential of fields, the identification and negation of susceptibility as well as deployment of resistance factors will be a significant milestone. With the increasing availability of cutting-edge tools, we are well-positioned to efficiently deploy these strategies in the previously neglected legumes, making a promising step forward toward sustainable disease management.

## Footnotes

^1^ https://www.pgro.org/downloads/LabPrices2024.pdf

## Supplementary material

Supplementary Table 1 Descriptive summaries of previously identified resistance factors plotted in Figure 3. Genetic factors involved in resistance against various root pathogens of pea are discussed in Section 6.1. The majority of these include QTLs identified through RIL mapping populations, however, increasingly, GWAS is also utilized. To approximate these intervals, sequences of genetic markers delimiting these intervals were obtained from various sources (Tayeh et al., 2015; Duarte et al., 2014; Boutet et al., 2016; Loridon et al., 2005). The physical locations of these markers were obtained on the ZW6 assembly using BLAST+2.2.28 (Camacho et al., 2009). For *P. ultimum* resistance, QTL locations were inferred from orthologous soybean marker genes. Additionally, the locations of pathogenesis-related genes involved in nonhost-responses were obtained from NCBI Gene search (Gene [Internet], 2004).
